# CANCER: First Combined Analysis from INTERPHONE Inconclusive

**Published:** 2010-07

**Authors:** Kellyn S. Betts

**Affiliations:** **Kellyn S. Betts** has written about environmental contaminants, hazards, and technology for solving environmental problems for publications including *EHP* and *Environmental Science & Technology* for more than a dozen years

Long-awaited results of the largest effort yet to investigate whether cell phone use contributes to brain cancers are finally available.^1^ But the May report, the first combined analysis of results from the International Agency for Research on Cancer’s (IARC) $24-million INTERPHONE study, is inconclusive, stating that “suggestions of an increased risk of glioma at the highest exposure levels, but biases and errors prevent a causal interpretation.”

The interview-based case–control INTERPHONE study was the collaborative effort of 48 researchers from 13 nations. It began in 2000 and included more than 14,000 participants, among them 2,765 glioma and 2,425 meningioma cases and matched controls^2^ (the current analysis included 2,708 glioma and 2,409 meningioma cases).No other studies have included as many exposed cases, particularly of long-term and heavy users of cell phones.

A major challenge the researchers faced in interpreting the data was the high refusal rates among controls—that is, controls were successfully contacted but declined to give the information sought—says the study’s principal investigator, Elisabeth Cardis, now of the Centre for Research in Environmental Epidemiology in Barcelona, Spain. “This resulted in mobile phone users being overrepresented among controls,” Cardis explains. The vast majority of the study’s risk estimates are below 1, which suggests there might have been a selection bias in amassing the study population, she says.

Additionally, cell phone usage patterns have changed significantly in the decade since INTERPHONE began. “Most of the users in the study had relatively low use compared to today’s use,” Cardis points out. The usage by people in the study’s highest cumulative call time group corresponds to about half an hour a day for a period of 10 years or more, which is “pretty normal or even light use today,” she says. At the same time, concerns over recall bias also made the data hard to interpret. For example, some cases—but no controls—claimed to spend 12 or more hours a day on their cell phone.

Besides the brain tumors assessed in the current study, INTERPHONE also evaluated correlations between cell phone use and tumors of the acoustic nerve and the parotid salivary gland. These two tumor types will be the focus of future reports, Cardis says.

The period of exposure for all of the subjects included in INTERPHONE is relatively short for assessing a causative link to a cancer, according to a commentary published alongside the study.^3^ Cell phone use began in the 1980s but was not widespread until the mid-1990s, wrote authors Rodolfo Saracci of Italy’s National Research Council in Pisa and Jonathan Samet of the University of Southern California’s Department of Preventive Medicine. “None of . . . today’s established carcinogens, including tobacco, could have been firmly identified as increasing risk in the first 10 years or so since first exposure,” explained Saracci and Samet. “Ionizing radiation is a recognized cause of brain tumors, but except for rare instances the radiation-induced cases occur on average after 10–20 years since the time of first exposure.” The authors conclude, therefore, that “observing no increase in risk would be reassuring but only to a limited extent.”

Publication of the first results from INTERPHONE was originally expected in 2006. Cardis says the report was delayed because of the large research team’s difficulties in interpreting the results. “The entire study group and all of the coauthors . . . spent a lot of time conducting hundreds of additional analyses, reviewing the analyses, and trying to understand the potential biases of the study,” she says. “We’ve conducted about every analysis that we could think to do.”

One of the analyses that did not make it into the main text of the report is Appendix 2, which is mentioned in Saracci and Samet’s commentary. Published only online as supplementary material, it presents an alternative analysis that suggests an increase in glioma among subjects in the top 10% of cumulative call time. The alternative analysis compared the incidence of glioma in the most highly exposed subjects to that in study subjects who had the lowest amount of exposure among regular cell phone users. In contrast, the primary analysis compared the incidence of glioma in the highly exposed group to the incidence among subjects who reported that they rarely or never used cell phones at all.

This approach—which accounts for the possibility that cell phone radiation exposure is not the only potential risk factor that differs between people who regularly use cell phones and people who don’t—is common in occupational epidemiology. However, some INTERPHONE investigators believed the analysis would be inappropriate if the main reason for the decreased odds ratios observed in the study was not selection bias. “We have legitimate differences in the interpretation of these results and the value of this analysis,” Cardis says.

IARC director Christopher Wild says, “Observations at the highest level of cumulative call time and the changing patterns of mobile phone use since the period studied by INTERPHONE, particularly in young people, mean that further investigation of mobile phone use and brain cancer risk is merited.” John Walls, vice president of public affairs for CTIA-The Wireless Association®, which represents the cell phone industry, says, “The possible effects of long-term heavy use of mobile phones require further investigation.”

Three important new studies are already under way to collect more data. The first is an animal study being conducted by the National Toxicology Program to assess the effects of long-term exposure to radiofrequency energy in rats and mice.^4^ The study allows for precise control over the exposure, as well as a “thorough evaluation for the presence of tumors, not just of the brain, but throughout the entire body,” says program associate director John Bucher.

The other two studies are epidemiologic. The case–control MOBI-KIDS study was launched last year in 13 countries to investigate potential risk factors for brain tumors in children, including cell phone use.^5^ Children’s rates of brain cancers have been rising in recent years, according to the study’s organizers, who hope to recruit approximately 2,000 brain cancer patients and matched controls. The COSMOS cohort study, launched in April with the specific goal of studying health effects of cell phone use, aims to recruit more than 250,000 people in five European countries and follow them for up to 30 years.^6^

## Figures and Tables

**Figure:**
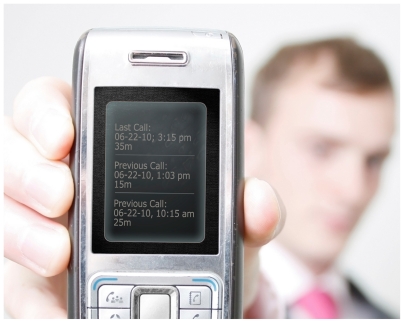
In the most recent industry figures from CTIA,^7^ U.S. cell phone users logged about 2.3 trillion minutes of use per year, but many users are reporting an increase in text messages over calls.
